# Creatinine, Diet, Micronutrients, and Arsenic Methylation in West Bengal, India

**DOI:** 10.1289/ehp.1003393

**Published:** 2011-06-07

**Authors:** Arin Basu, Soma Mitra, Joyce Chung, D.N. Guha Mazumder, Nilima Ghosh, David Kalman, Ondine S. von Ehrenstein, Craig Steinmaus, Jane Liaw, Allan H. Smith

**Affiliations:** 1 Arsenic Health Effects Research Group, School of Public Health, University of California, Berkeley, California, USA; 2 Health Sciences Centre, University of Canterbury, Christchurch, New Zealand; 3 School of Human Sciences, London Metropolitan University, London, United Kingdom; 4 Veteran’s Administration, Palo Alto Health Care System, Palo Alto, California, USA; 5 Department of Gastroenterology, Institute of Post Graduate Medical Education and Research, Kolkata, India; 6 Department of Environmental Health, University of Washington, Seattle, Washington, USA; 7 School of Public Health, University of California, Los Angeles, California, USA; 8 Office of Environmental Health Hazard Assessment, California Environmental Protection Agency, Oakland, California, USA

**Keywords:** arsenic, creatinine, diet, India, methylation, micronutrients, West Bengal

## Abstract

Background: Ingested inorganic arsenic (InAs) is methylated to monomethylated (MMA) and dimethylated metabolites (DMA). Methylation may have an important role in arsenic toxicity, because the monomethylated trivalent metabolite [MMA(III)] is highly toxic.

Objectives: We assessed the relationship of creatinine and nutrition—using dietary intake and blood concentrations of micronutrients—with arsenic metabolism, as reflected in the proportions of InAS, MMA, and DMA in urine, in the first study that incorporated both dietary and micronutrient data.

Methods: We studied methylation patterns and nutritional factors in 405 persons who were selected from a cross-sectional survey of 7,638 people in an arsenic-exposed population in West Bengal, India. We assessed associations of urine creatinine and nutritional factors (19 dietary intake variables and 16 blood micronutrients) with arsenic metabolites in urine.

Results: Urinary creatinine had the strongest relationship with overall arsenic methylation to DMA. Those with the highest urinary creatinine concentrations had 7.2% more arsenic as DMA compared with those with low creatinine (*p <* 0.001). Animal fat intake had the strongest relationship with MMA% (highest tertile animal fat intake had 2.3% more arsenic as MMA, *p <* 0.001). Low serum selenium and low folate were also associated with increased MMA%.

Conclusions: Urine creatinine concentration was the strongest biological marker of arsenic methylation efficiency, and therefore should not be used to adjust for urine concentration in arsenic studies. The new finding that animal fat intake has a positive relationship with MMA% warrants further assessment in other studies. Increased MMA% was also associated, to a lesser extent, with low serum selenium and folate.

Arsenic in drinking water is a major public health problem in the Bengal delta, affecting millions of people in the West Bengal state of India and adjoining areas in Bangladesh. In view of widespread poor nutrition prevalent in West Bengal and Bangladesh, it is important to identify dietary factors that might increase arsenic toxicity or metabolism. We have already reported on the relationship of dietary constituents and blood micronutrients with arsenic-caused skin lesions in West Bengal (Chung et al. 2006; Mitra et al. 2004). Here, we report the findings concerning 19 dietary intake constituents, 16 blood micronutrients, and urine creatinine with arsenic methylation patterns in urine, in what we believe is the first comprehensive joint assessment of dietary intake and micronutrients. For brevity, we refer to the dietary intake factors, along with the blood micronutrients, as “nutritional factors.”

Nutritional factors may play important roles in methylation of inorganic arsenic (InAs), and the results of numerous studies have shown that methylation affects the toxicity of ingested InAs. In drinking water, InAs exists in trivalent [As(III)] and pentavalent [As(V)] forms. After ingestion, As(V) is reduced to As(III), followed by sequential methylation and reduction reaction steps resulting in formation of trivalent and pentavalent methylated arsenicals—first monomethyl acids [MMA(III) and MMA(V)] and subsequently trivalent and pentavalent dimethyl acids [DMA(III) and DMA(V), respectively. However, this process is incomplete, and populations show variations in the proportions of urinary InAs and the methylated arsenical compounds (Aposhian 1997; Concha et al. 2002; Drobna et al. 2004; Healy et al. 1997; Hopenhayn-Rich et al. 1996a, 1996b; Hopenhayn et al. 2003; Hughes 2002).

Isolation and characterization of MMA(III) indicates that the first step of methylation that produces MMA should be considered an activation step. Laboratory studies have shown that the trivalent forms of MMA and DMA are much more toxic than the pentavalent forms, and *in vitro* evidence suggests that MMA(III), in particular, may be more toxic than trivalent InAs [InAs(III)] (Cullen and Reimer 1989; Lin et al. 1999, 2001; Mass et al. 2001; Petrick et al. 2000; Styblo et al. 1999, 2000; Styblo and Thomas 1997). The conversion of MMA(III) to DMA(V) in a second methylation step then reduces toxicity. Epidemiological studies have found evidence of increased risks of skin and bladder cancer among persons with higher proportions of MMA in their urine (Steinmaus et al. 2003). Therefore, factors that influence arsenic methylation may be important determinants of susceptibility to health effects resulting from exposure to InAs in drinking water. We were also interested in urine creatinine, because it is frequently used to adjust for the concentration of urine, something that would introduce bias if creatinine were related to arsenic methylation.

## Methods

*Study participants.* This study involved 405 participants selected from a study base of 7,683 individuals living in the South 24-Parganas district of West Bengal, India, who were identified in a 1995 through 1996 cross-sectional survey (Guha Mazumder et al. 1998). Participants had been selected for a case–control study of skin lesions caused by arsenic involving 192 cases and 213 controls (Haque et al. 2003), whose primary sources of drinking water contained < 500 µg/L InAs [*n* = 4,185 (2,160 females and 2,025 males)].

Cases had positive skin-lesion classifications, with either hyperpigmentation (mottled, dark-brown pigmentation bilaterally distributed on the trunk), keratoses (diffuse thickening of palms or soles, with or without nodules), or both conditions at the time of the survey. Of the 7,683 individuals in the base population, we randomly selected controls from a subset of 4,185 individuals, with drinking water concentrations of arsenic < 500 µg/L; controls were matched to cases by sex and by age within 4 years. The study protocol was approved by the institutional review boards of the Institute of Post Graduate Medical Education and Research, Kolkata, India, and the University of California–Berkeley, Berkeley, California. Informed consent was obtained from all participants.

In this investigation, we assessed dietary intake and measured blood micronutrients and methylated arsenic species in urine samples for pooled cases and controls. Cases with skin lesions were included after adjusting for possible distortion of associations between the nutritional factors and arsenic methylation patterns by including an indicator variable for skin lesions in the analyses.

In India, socioeconomic status is commonly measured by type of dwelling, which is correlated with household economic status (Mishra et al. 1999). In this study, we determined socioeconomic status based on the materials used to construct the house where the respondent lived. We considered three types of houses: pucca houses, built with high-quality materials such as bricks or concrete; semipucca houses, constructed partly with clay and bricks; and kacha (mud houses). We classified educational status as nonformal (participant never attended school), primary education (up to 4 years of education), high school education (between 8 and 12 years of education), and beyond high school education or tertiary education (> 12 years of formal education).

*Assessment of dietary intake and blood micronutrients.* We ascertained food intake for each participant with a detailed questionnaire based primarily on 24-hr recall. The methods used for dietary assessment have been described elsewhere (Mitra et al. 2004). In brief, the most senior woman, who in this population directed the preparation of food for the family, was interviewed and questioned about each meal from lunch the previous day through breakfast on the day of the interview. The volume of each cooked food was assessed by asking the senior woman to estimate these volumes using standard cups and plates. Standard-sized spoons were used to assess the intake of sugar and oil. We asked about weekly consumptions of meat, fish, eggs, milk, and fruit, because these items were not typically consumed on a daily basis. The 1-week intake of these food items was then divided by 7 to compute the mean intake per day. We calculated total 24-hr intake of the following nutrients using a spreadsheet program based on food composition tables (listed here in alphabetical order): animal fat, animal protein, calcium, carbohydrate, carotene, fiber, folate, iron, niacin, phosphorus, retinol, thiamin, vegetable fat, vegetable protein, vitamin B_6_, vitamin B_12_, vitamin C, and zinc (Mitra et al. 2004). We adjusted each dietary variable for individual total calorie intake by dividing total daily dietary intake by total calorie intake.

Field team physicians interviewed participants using a structured questionnaire, conducted a general examination, and obtained blood samples from each participant when they were visited in their homes. We have previously presented detailed information concerning storage and analysis of blood samples (Chung et al. 2006). In brief, nonfasting blood samples were collected and stored in an ice chest in the field. Aliquots were prepared within 24 hr, frozen at –20°C in India, and later transported to the United States on dry ice, where they were stored at –70°C until laboratory analysis. Pacific Biometrics (Seattle, WA, USA) conducted most of the serum and plasma analyses for the micronutrients and biochemical indicators or in some instances arranged for them to be done at a different laboratory. Plasma measurements included homocysteine, glutathione, cysteine, methionine, vitamin B_6_, retinol (vitamin A), alpha-tocopherol (vitamin E), alpha-carotene, beta-carotene, lycopene, lutein-zeaxanthin, and beta-cryptoxanthine (Chung et al. 2006). Serum measurements included glucose, cholesterol, vitamin B_12_ (cobalamin), folate, transthyretin, and selenium.

*Measurement of urinary arsenic and creatinine.* Because urine is the primary route of arsenic excretion, the proportions of the various arsenic metabolites in urine are commonly used as an indicator of the degree to which a person can methylate ingested InAs (National Research Council 2001). Spot urine samples were collected from each participant and stored frozen for later analysis at the Trace Organics Analysis Laboratory (University of Washington, Department of Environmental and Occupational Health Sciences, Seattle, Washington). The urinary concentrations of arsenic were measured using hydride generation/cryogenic concentration, atomic fluorescence detection, using an Excalibur AFS detector (PS Analytical, Inc., Deerfield Beach, FL). In this technique, InAs, MMA, and DMA were reduced to the corresponding arsine in a batch reactor, using sodium borohydride in 5-mL samples. The volatile reduction products (arsenic, methyl arsine, and dimethylarsine) were removed by sparging with helium. Entrained arsines were concentrated in a chromosorb-filled cryogenic trap at liquid nitrogen temperatures until all arsine-forming arsenic in the sample had reacted. The cryotrap was then allowed to warm, and the collected arsines were separated on the basis of differential volatilization. The separated volatile arsenic species were detected with a hydrogen microburner combustion cell to convert arsines to elemental arsenic. To prevent interference by other compounds, each urine sample was acidified with 2 M HCl and allowed to sit for at least 4 hr prior to speciation analysis. Total arsenic was determined by atomic fluorescence spectrometry with flow injection analysis (Excalibur AFS, PS Analytical) on a portion of the urine sample that had been mineralized with hydrochloric acid and peroxide; the result was compared with the sum of the species detected. If a significant amount of arsenic remained undetected, additional digestion or assay for arsenobetaine was performed. Detection limits for InAs, MMA, and DMA were 0.5, 1, and 2 µg/L, respectively. Concentrations below the detection limit were set at one-half the detection limit. The methods detected both oxidation states for each form of arsenic (inorganic, monomethyl, dimethyl) and provided a single result for each methylation level. Urinary arsenic concentrations were measured in micrograms per liter of urine; percentages of InAs (InAs%), MMA (MMA%), and DMA (DMA%) were calculated using the sum of arsenic species detected as the denominators. The creatinine concentrations in urine were determined kinetically by a clinical autoanalyzer using the Jaffe reaction (Larsen 1972).

*Statistical methods.* The relationships between demographic variables and urinary arsenic methylation patterns were first assessed in simple stratified analyses. Nutritional factors and urine creatinine were divided into tertiles, and we compared arsenic methylation patterns in the highest tertile of each factor with patterns in the lowest tertile. We conducted *t*-tests of the differences in mean urinary InAs% between the highest and lowest tertiles of each nutritional factor. Nutritional factors were then ranked, starting with the nutritional factor associated with the largest absolute mean difference in InAs% between its highest and lowest tertiles. Nutritional variables with the highest mean differences were then entered into a series of multiple linear regression analyses, as explained below. This series of steps was repeated for MMA% and DMA%.

In the multiple linear regression models, InAs%, MMA%, and DMA% were treated as response variables, and tertiles of selected dietary or micronutrient variables were the main explanatory variables. The lowest tertile of the dietary or micronutrient variable was treated as the reference category, and dummy variables were created for the middle and highest tertile for each nutritional variable. We used step-up methods, starting with the strongest predictors found in the univariate analyses. The beta coefficients corresponding to the highest tertile of the diet or micronutrient gives an effect measure of the potential impact of each nutrient on the specific urinary methylated arsenical. For example, a beta coefficient of –5 in the model of InAs% for a particular nutrient would indicate 5% difference (e.g., 20% InAs% for the high tertile compared with 25% InAs% for the low tertile), with adjustment for the effect of all other variables specified in the model. This represents the independent effect of the nutrient on the specified urinary methylated arsenical. In addition to nutritional factors, the other variables in the model were age, sex, housing, education, presence or absence of skin lesions, body mass index (BMI), and total urinary arsenic. Housing and education were modeled as categorical variables, whereas age, BMI, and urinary arsenic were modeled as continuous variables.

The important advantages of using tertiles of nutritional factors in the analyses, rather than continuous variables, include the fact that there is no implicit assumption about the functional form of any dose–response relationship. In addition, outliers often have undue influence on regression analyses, and dividing into tertiles avoids this problem, as outliers are merely one member of the tertile to which they belong. However, we repeated the analyses that produced the key findings using continuous variable regression and obtained similar results. *p*-Values were based on chi-square tests of trend for the following ordinal variables: categories of age, education, type of housing, urinary arsenic, and BMI (Table 1).

**Table 1:** Distribution of the percentage of urinary total arsenic in inorganic form (InAs%), monomethylated (MMA%), and dimethylated (DMA%), according to other factors for the 405 participants.

Characteristics	*n *(%)		InAs%	MMA%	DMA%
Age (years)									
< 15		28	(6.9)		20.4		6.8		72.8
15–29		91	(22.5)		23.3		8.4		68.3
30–44		135	(33.4)		23.6		8.3		68.1
45–59		89	(21.9)		24.8		7.9		67.3
≥ 60		62	(15.3)		19.5		9.3		71.2
*pa*					0.19		0.06		0.14
Sex									
Female		154	(38.2)		24.5		7.0		68.5
Male		251	(61.8)		22.2		8.9		68.9
*pb*					0.05		0.01		0.2
Education									
Nonformal		117	(28.9)		22.5		8.2		69.3
Primary		200	(49.5)		23.5		8.2		68.3
Secondary		68	(16.8)		22.8		8.0		69.2
Tertiary		20	(4.9)		22.4		8.3		69.3
*pa*					0.47		0.48		0.46
House type									
Kacha		205	(50.6)		22.6		8.3		69.1
Semipucca		138	(34.1)		24.1		7.9		68.1
Pucca		58	(14.3)		22.3		8.5		69.2
Missing data		4	(0.9)						
*pa*					0.32		0.25		0.4
Skin lesions									
Present		213	(52.5)		21.2		8.3		70.4
Absent		192	(47.4)		24.7		8.1		67.2
*pb*					0.02		0.56		0.03
BMI									
< 16.9		122	(30.1)		23.5		8.0		68.5
16.9–19.3		121	(29.8)		21.3		8.7		70.1
> 19.3		126	(31.1)		22.2		8.4		69.4
Missing		36	(8.9)						
*pa*					0.52		0.42		0.73
Total urinary arsenic (µg/L)									
< 14.9		134	(33.1)		20.4		8.5		71.1
14.9–56.3		133	(32.8)		22.5		7.8		69.6
> 56.3		138	(34.1)		21.5		8.5		69.9
*pa*					0.55		0.48		0.71
**a***p*-Value from test for trend. **b***p*-Value from *t*-test.									

## Results

The distribution of participants according to age, sex, education, and quality of housing is shown in Table 1. Men had lower urinary InAs% (men, 22.2%; women, 24.5%; *p* = 0.05) and higher MMA% than did women (men, 8.9%; women, 7.0%; *p* = 0.01). Those with arsenic-caused skin lesions had lower urinary InAs% (21.2% vs. 24.7% for those without skin lesions; *p* = 0.02), and higher urinary DMA% (70.4% vs. 67.2%; *p* = 0.03). Table 2 presents the mean ± SD of the dietary factors intake per day and the mean concentrations of blood micronutrients.

**Table 2:** Mean intake per day of dietary factors, mean plasma and serum concentrations of micronutrients, and mean urine creatinine concentrations, with SDs for the 405 participants.

Dietary intake/micronutrient concentration	Mean ± SD	
Diet survey		
Calcium (mg/day)	484	± 332
Carbohydrate (g/day)	448	± 161
Carotene (µg/day)	3,546	± 7,161
Energy (kJ/day)	9,204	± 3,091
Fat, animal (g/day)	3.91	± 4.24
Fat, vegetable (g/day)	21.8	± 14.9
Fiber (g/day)	5.3	± 3.47
Folate (µg/day)	164	± 102
Iron (mg/day)	13.9	± 7.0
Niacin (mg/day)	22	± 8.1
Phosphorus (mg/day)	1,130	± 435
Protein, animal (g/day)	9.7	± 7.14
Protein, vegetable (g/day)	45	± 17.1
Retinol (µg/day)	52.2	± 66.1
Riboflavin (mg/day)	0.6	± 0.3
Thiamin (mg/day)	1.49	± 0.6
Vitamin B_6_ (mg/day)	1.3	± 0.5
Vitamin C (mg/day)	111	± 115
Zinc (mg/day)	9.2	± 3.3
Plasma analysis		
Alphatocopherol (µg/dL)	636	± 214
Beta-carotene (µg/dL)	83.6	± 97.3
Beta-cryptoxanthine (µg/dL)	5.74	± 5.6
Homocysteine (µM/L)	14.8	± 8.1
Lutein-zeaxanthine (µg/dL)	66.5	± 30.6
Lycopene (µg/dL)	3.3	± 4.9
Methionine (µM/L)	19.4	± 5.7
Retinol (µM/dL)	34.1	± 10.9
Transthyretin (mg/L)	237	± 53.1
Vitamin B_6_ (nmol/L)	40.8	± 45.3
Serum analysis		
Cholesterol (mg/dL)	155	± 35.4
Cysteine (µM/L)	215	± 37.5
Folate (ng/mL)	3.4	± 2.8
Selenium (µM/L)	1.2	± 0.4
Total glutathione (µM/L)	6.3	± 5.6
Vitamin B_12_ (pg/mL)	435	± 315
Urine		
Urine creatinine (mg/L)	622	± 531.8

The differences in mean urinary InAs%, MMA%, and DMA% between those in the highest and lowest tertiles of each dietary intake factor and blood micronutrient are presented in Table 3, ranked in order of the magnitude of the absolute differences, excluding those factors having a *p*-value > 0.2. The largest difference in urinary InAs% was for urinary creatinine, with those in the highest tertile of creatinine having 19.8 InAs% compared with those in the lowest tertile, who had 27.7% (difference 7.8%; *p* ≤ 0.001). Those in the highest tertile of plasma lycopene also had lower InAs% (20.5% compared with 26.3%; *p =* 0.01). The largest differences in MMA% were for dietary animal fat, plasma retinol, plasma homocysteine, dietary retinol, and dietary animal protein, followed by serum folate and urinary creatinine, all of which were associated with *p*-values < 0.01. The largest difference (2.3%) was for animal fat (*p <* 0.001). When we repeated the analysis with animal fat as a continuous variable, the result was the same (*p* < 0.001). The strongest association with DMA% was for urine creatinine, with those in the highest tertile of creatinine having a DMA% of 71.4% compared with those in the lowest tertile, who had DMA% of 64.2% (difference 7.2%; *p* < 0.001). When we repeated the analysis with urine creatinine as a continuous variable, the *p*-value was the same (< 0.001).

**Table 3:** Association between dietary variables, serum levels of micronutrients, and urinary creatinine as assessed by comparing the mean arsenic metabolite percent between the lowest (T1) and highest (T3) tertiles of each factor.

Metabolite	Nutritional factor	T1	T3	Difference	*p*-Value*a*
InAs%		Urine creatinine		27.7		19.8		–7.8		0.001
		Plasma lycopene		26.3		20.5		–5.9		0.01
		Dietary riboflavin		20.9		25.2		4.3		0.04
		Serum selenium		19.6		23.7		4.1		0.03
		Serum folate		20.4		24.3		3.9		0.04
		Plasma vitamin B_6_		23.5		20.4		–3.1		0.14
		Dietary calcium		23.0		25.9		2.9		0.19
		Plasma lutein-zeaxanthine		23.9		21.0		–2.9		0.14
MMA%		Dietary animal fat		7.0		9.3		2.3		0.001
		Plasma retinol		7.3		9.2		1.9		0.001
		Plasma homocysteine		7.3		9.2		1.9		0.002
		Dietary retinol		7.3		9.1		1.8		0.004
		Dietary animal protein		7.0		8.7		1.7		0.003
		Serum folate		9.6		8.0		–1.7		0.007
		Urine creatinine		7.2		8.7		1.5		0.004
		Serum selenium		9.2		7.7		–1.5		0.02
		Plasma alpha-tocopherol		8.0		9.1		1.1		0.07
		Plasma beta-cryptoxanthine		7.3		8.4		1.1		0.07
		Plasma vitamin B_6_		7.7		8.7		1.0		0.11
		Plasma methionine		7.8		8.8		0.9		0.11
		Dietary phosphorus		7.3		8.1		0.9		0.15
		Dietary carbohydrate		8.6		7.8		–0.8		0.15
DMA%		Urine creatinine		64.2		71.4		7.2		0.001
		Dietary riboflavin		70.5		65.9		–4.6		0.03
		Plasma lycopene		65.4		69.6		4.3		0.07
		Plasma lutein-zeaxanthine		65.9		69.9		4.0		0.06
The table is limited to variables with *p* < 0.20. **a***p*-Value from the *t*-test of the mean difference.										

The results of the multivariate analysis are presented in Table 4, using step-up methods to explore the findings. Covariates included in all models were age, sex, housing, education, skin lesion status, total urinary arsenic, and BMI. The first model for InAs (InAs1) included creatinine and lycopene. In the univariate analysis (Table 3), high creatinine was associated with a lower InAs%, with a difference of 7.8%. With the covariates and lycopene in the model, the size of this difference is reduced from 7.8% to 4.6%, and lycopene becomes the more important variable both in terms of the difference in magnitude (4.9%) and *p*-values (0.03 vs. 0.08, Table 4). The association with creatinine was further reduced when riboflavin was added to the model.

**Table 4:** Results of multivariate analysis of nutritional factors measured in diet (d), plasma (p), serum (s), and urine (u) with indicators of arsenic methylation InAs%, MMA%, and DMA%. The beta coefficients are for the highest tertile of each nutritional factor with the lowest tertile as the reference category.

Model	Nutritional factor	Beta coefficient	Lower CI limit	Upper CI limit	*p*-Value
InAs% models										
InAs1		Creatinine (u)		–4.6		–11.0		1.8		0.08
		Lycopene (p)		–4.9		–9.4		–0.4		0.03
InAs2		Creatinine (u)		–4.1		–9.0		0.8		0.14
		Lycopene (p)		–6.2		–11.2		–1.2		0.02
		Riboflavin (d)		5.9		0.9		10.9		0.02
InAs3		Creatinine (u)		–3.4		–8.0		1.2		0.20
		Lycopene (p)		–5.3		–10.2		–0.4		0.04
		Riboflavin (d)		4.6		–0.4		9.6		0.07
		Selenium (s)		2.5		–2.6		7.6		0.35
MMA% models										
MMA1		Animal fat (d)		2.0		0.8		3.3		0.003
		Retinol (p)		1.8		0.5		3.1		0.005
MMA2		Animal fat (d)		1.9		0.6		3.2		0.01
		Retinol (p)		1.7		0.4		3.1		0.02
		Homocysteine (p)		1.9		0.6		3.3		0.01
MMA3		Animal fat (d)		1.9		0.5		3.3		0.003
		Retinol (p)		1.6		0.2		3.0		0.03
		Homocysteine (p)		1.1		–0.4		2.6		0.14
		Folate (s)		–2.1		–3.6		–0.6		0.03
MMA4		Animal fat (d)		2.3		0.9		3.7		0.001
		Retinol (p)		2.1		0.7		3.5		0.002
		Selenium (s)		–2.2		–3.6		–0.8		0.001
MMA5		Animal fat (d)		2.5		1.1		3.9		0.001
		Retinol (p)		2.0		0.6		3.4		0.006
		Folate (s)		–1.1		–2.6		0.4		0.15
		Selenium (s)		–2.3		–3.8		–0.9		0.002
DMA% models										
DMA1		Creatinine (u)		5.4		0.3		10.5		0.04
		Riboflavin (d)		–3.3		–8.0		1.4		0.17
DMA2		Creatinine (u)		5.7		0.2		11.2		0.04
		Riboflavin (d)		–3.8		–8.9		1.3		0.15
		Lycopene (p)		3.9		–1.2		9.0		0.13
Covariates in all models were age (continuous variable), sex, housing, education, skin lesion status, total urinary arsenic (as a continuous variable), and BMI (as a continuous variable).										

Table 4 also presents five models (MMA1–MMA5) for MMA. In the univariate analysis, there were eight factors that had strong relationships with MMA% (*p* ≤ 0.02). In the multivariate models, animal fat remains important in all models, with high animal fat intake related to a high MMA%. To a lesser extent, high plasma retinol was also associated with high MMA%, whereas high serum selenium was associated with a lower MMA%. Two DMA models are given in Table 4; the strongest relationship was with urinary creatinine, with a high urine creatinine being associated with a high DMA%.

## Discussion

Diet and arsenic methylation, and blood micronutrients and arsenic methylation, have previously been assessed separately (Hall et al. 2009; Heck et al. 2007, 2009; Huang et al. 2009; Steinmaus et al. 2005). We believe this is the first comprehensive study of arsenic methylation including both dietary and micronutrient variables, along with urinary creatinine, considered together in the same analysis. New findings to emerge from this work include the fact that urinary creatinine was the strongest predictor of DMA%, with a high urinary creatinine being associated with more InAs being fully methylated to DMA. Once urinary creatinine was taken into account, no nutritional factor was a significant predictor of DMA%. A high urinary creatinine was associated in the opposite direction with InAs%, which had a lower percentage in urine when creatinine was high. This association for InAs% decreased when lycopene and riboflavin were included in the model, with lycopene becoming the strongest predictor variable in that a high lycopene concentration was associated with a low InAs%. One previous study suggested that low plasma lycopene was associated with reduced arsenic methylation capacity (Hsueh et al. 2009). However, as can be seen in Table 4, we find that full methylation to DMA was more strongly related to urinary creatinine than to lycopene.

The relationship between creatinine and arsenic methylation has been reported previously as an aside, without noting that it is the most important predictor nor being able to compare it with multiple nutritional factors as we have done here. In the past, urinary creatinine has usually been used to normalize for urine concentration, to compensate for variation in diuresis. However, problems with doing so have been identified (Barr et al. 2005), in particular for studies of arsenic (Nermell et al. 2008; Steinmaus et al. 2009). Gamble et al. reported a strong correlation between urinary creatinine and DMA% (*p <* 0.001) (Gamble et al. 2005), and Nermell et al. (2008) noted that urinary creatinine was correlated mainly with DMA. One recent study found that low urinary creatinine was associated with arsenic-induced skin lesions (Pilsner et al. 2009). It is noteworthy that the formation of creatinine from methylation of guanidinoacetate accounts for approximately 75% of all folate-dependent transmethylation reactions (Barr et al. 2005; Mudd and Poole 1975).

Another new finding was that animal fat in the diet was by far the strongest predictor of MMA%, with a high intake of animal fat being associated with a high MMA%. Some investigators have thought that a high MMA% would be due to an increase in the first step of methylation from InAs% to MMA. However, because 85% of InAs, on average, is methylated, at least to MMA, and the large majority ends up as DMA, increases in MMA% are more likely due to a reduction in the second step of methylation from MMA to DMA. We do not know why a high intake of animal fat would reduce the second step of methylation from MMA to DMA. One possibility is that animal fat intake is related to some unknown confounding factor. The only published study we could find that mentioned fat intake and methylation was a study of 66 adults who were kept on a fixed diet (Poirier et al. 2001). The investigators reported that during the experimental period of the study, when dietary fat intake was higher than during the usual diet, blood *S-*adenosylmethionine levels were inversely correlated with fat intake. The positive association between dietary animal fat and MMA% is a new finding not reported elsewhere. In a study on dietary intake and arsenic methylation based on a U.S. population (*n* = 30), Steinmaus et al. (2005) did not find any relationship between fat intake and MMA% in urine. A possible reason why our findings were different from the U.S. study could be attributed to the different levels and patterns of nutrition between a U.S. population and this rural Indian population.

The inverse association between serum folate and selenium and MMA% is supported by findings from other epidemiological studies. Using data from a cross-sectional survey in a comparable Bangladeshi population (*n* = 300), Gamble et al. reported that serum folate was inversely associated with InAs% (Spearman *r* = –0.12; *p* < 0.05) and MMA% (*r* = –0.12; *p* = 0.04) and was positively associated with serum homocysteine (*r* = 0.21; *p* < 0.05) and urinary DMA% (*r* = 0.21; *p* < 0.001) (Gamble et al. 2005). Gamble and colleagues reported univariate correlation estimates of associations and did not report results of multivariate associations after adjustment of possible confounding variables. On the same population, Gamble et al. (2005) conducted a follow-up double-blind randomized controlled trial (*n* = 200) that compared 12-week administrations of folate versus placebo and found that after 12 weeks of folic acid supplementation, the increase in the proportion of total urinary arsenic excreted as DMA in the folate group (72% at baseline and 79% after supplementation; *p <* 0.0001) was greater than that in the placebo group, as was the reduction in the proportions of total urinary arsenic excreted as MMA (13% and 10%, respectively; *p <* 0.0001) and as InAs (15% and 11%, respectively; *p <* 0.001). However, in this study, the intervention group and the placebo group were exposed to arsenic during the time of the trial (Gamble et al. 2006). Because this study was conducted among individuals concomitantly exposed to high concentrations of InAs, it cannot be inferred that folate supplementation would benefit individuals who were no longer exposed to arsenic in drinking water.

Studies have consistently reported higher MMA% in males than in females (National Research Council 1999). In the univariate comparison, the difference in MMA% between men and women was 1.9 (Table 1). This difference was not much less than for animal fat (2.3, Table 3). The difference between males and females may be explained in part by differences in animal fat intake and by differences in urinary creatinine.

Associations that might have been expected but were largely absent include homocysteine, vitamin B_12_ (cobalamin), and methionine. Homocysteine was strongly related to MMA% in univariate analysis, but the association became weaker in multivariate modeling when folate was added (Table 4). Heck et al. 2007 reported a relationship between cobalamin and increased MMA to InAs ratio, but we found no evidence of it in this study (MMA% for the lowest tertile of vitamin B_12_ = 8.0 and for the highest tertile MMA% = 8.1; *p =* 0.75).

Data for this study came from a cross-sectional survey. Information on the dietary variables was collected at the same time as blood was sampled for estimation of serum micronutrients, and urine was collected for estimation of urinary InAs%, MMA%, and DMA%. This provides a snapshot of interrelationships between diet, micronutrients, and arsenic methylation variables. Usually, the main limitation of cross-sectional studies pertains to inferring long-term outcomes from data obtained at one point in time. However, this limitation does not apply here, because it is reasonable to believe that arsenic methylation patterns would relate to current nutritional factors. In addition, it has been found that arsenic methylation efficiency in individuals is relatively stable over time (Concha et al. 2002). Another limitation is that because we did not have measures of urinary specific gravity, it was not possible to adjust for urine concentration. Our findings and those of others indicate that one should not use urinary creatinine to adjust for urine concentration in studies of arsenic, especially those involving speciation. However, it should be noted that the parameters of interest in this study were InAs%, MMA%, and DMA%. The proportion of arsenic in these various forms is unlikely to be related to urine concentration, even though the concentrations in absolute terms would be.

## Conclusions

This study revealed that urinary creatinine in the highest versus lowest tertile had a stronger association with arsenic methylation to DMA than did similar contrasts for any of the dietary or micronutrient factors assessed. This finding provides further evidence that urinary creatinine should not be used to adjust urine concentrations for diuresis effects. Further studies are needed to investigate the determinants of urine creatinine concentrations in arsenic-exposed populations, because such factors are also likely to influence arsenic methylation. The new findings that plasma lycopene had an inverse relationship with InAs% and that animal fat intake had a positive relationship with MMA% are new and warrant further assessment in other studies.
